# Propagating Cell-Membrane Waves Driven by Curved Activators of Actin Polymerization

**DOI:** 10.1371/journal.pone.0018635

**Published:** 2011-04-21

**Authors:** Barak Peleg, Andrea Disanza, Giorgio Scita, Nir Gov

**Affiliations:** 1 Department of Chemical Physics, the Weizmann Institute of Science, Rehovot, Israel; 2 IFOM, the FIRC Institute for Molecular Oncology Foundation, Milan, Italy; 3 Department of Medicine, Surgery and Dentistry, Università degli Studi di Milano, Milan, Italy; Université d'Evry val d'Essonne, France

## Abstract

Cells exhibit propagating membrane waves which involve the actin cytoskeleton. One type of such membranal waves are Circular Dorsal Ruffles (CDR) which are related to endocytosis and receptor internalization. Experimentally, CDRs have been associated with membrane bound activators of actin polymerization of concave shape. We present experimental evidence for the localization of convex membrane proteins in these structures, and their insensitivity to inhibition of myosin II contractility in immortalized mouse embryo fibroblasts cell cultures. These observations lead us to propose a theoretical model which explains the formation of these waves due to the interplay between complexes that contain activators of actin polymerization and membrane-bound curved proteins of both types of curvature (concave and convex). Our model predicts that the activity of both types of curved proteins is essential for sustaining propagating waves, which are abolished when one type of curved activator is removed. Within this model waves are initiated when the level of actin polymerization induced by the curved activators is higher than some threshold value, which allows the cell to control CDR formation. We demonstrate that the model can explain many features of CDRs, and give several testable predictions. This work demonstrates the importance of curved membrane proteins in organizing the actin cytoskeleton and cell shape.

## Introduction

Living cells have the ability to produce propagating waves on their membranes, which are traveling membrane undulations involving an accumulation of the actin cytoskeleton, that persist over microns and during minutes. Such membrane waves have been observed in a variety of cells, during cell spreading [1]–[3] and in response to excitation by soluble factors [4]. These waves are believed to play a role in cellular motility, probing of the surrounding matrix, endocytosis and internalization of membrane receptors [4]. In the damped liquid environment of the cell, these propagating waves are maintained by the constant supply of active forces from the cytoskeleton. The main type of active force at the membrane is the protrusive force due to the polymerization of actin filaments near the membrane.

The mechanisms responsible for these different waves are not well understood at present. Several theoretical models have been suggested to explain the propagation of actin waves on the membrane of cells [5], [6]. One kind of mechanism that was shown to drive membrane-cytoskeleton waves involves the recruitment to the membrane of actin polymerization by curved membrane proteins (activators). The coupling between the membrane shape and the protrusive force of actin polymerization was shown to produce damped waves when only concave activators are present [7]. In contrast, a model that was able to produce non-decaying waves relied on the addition of contractile forces produced by myosin II motors, in conjunction with only convex actin activators [8]. This model was shown to fit recent experiments [9], where myosin inhibition abolished the observed waves. Conversely, other types of membrane ruffles are insensitive to inhibition of actomyosin contractility or to the genetic removal of myosin II (Supporting movies 7 and 8 of [10]). In order to account for such waves that do not require myosin-driven contractility, we explore in this paper wether only using the protrusive forces of actin polymerization can give rise to non-decaying membrane-cytoskeleton waves. We indeed identify a new mechanism for such waves, based on the interplay between curved membrane proteins of both convex and concave shapes, and give a specific biological example where it may apply.

## Results

### Experimental Results

**Figure 1:**
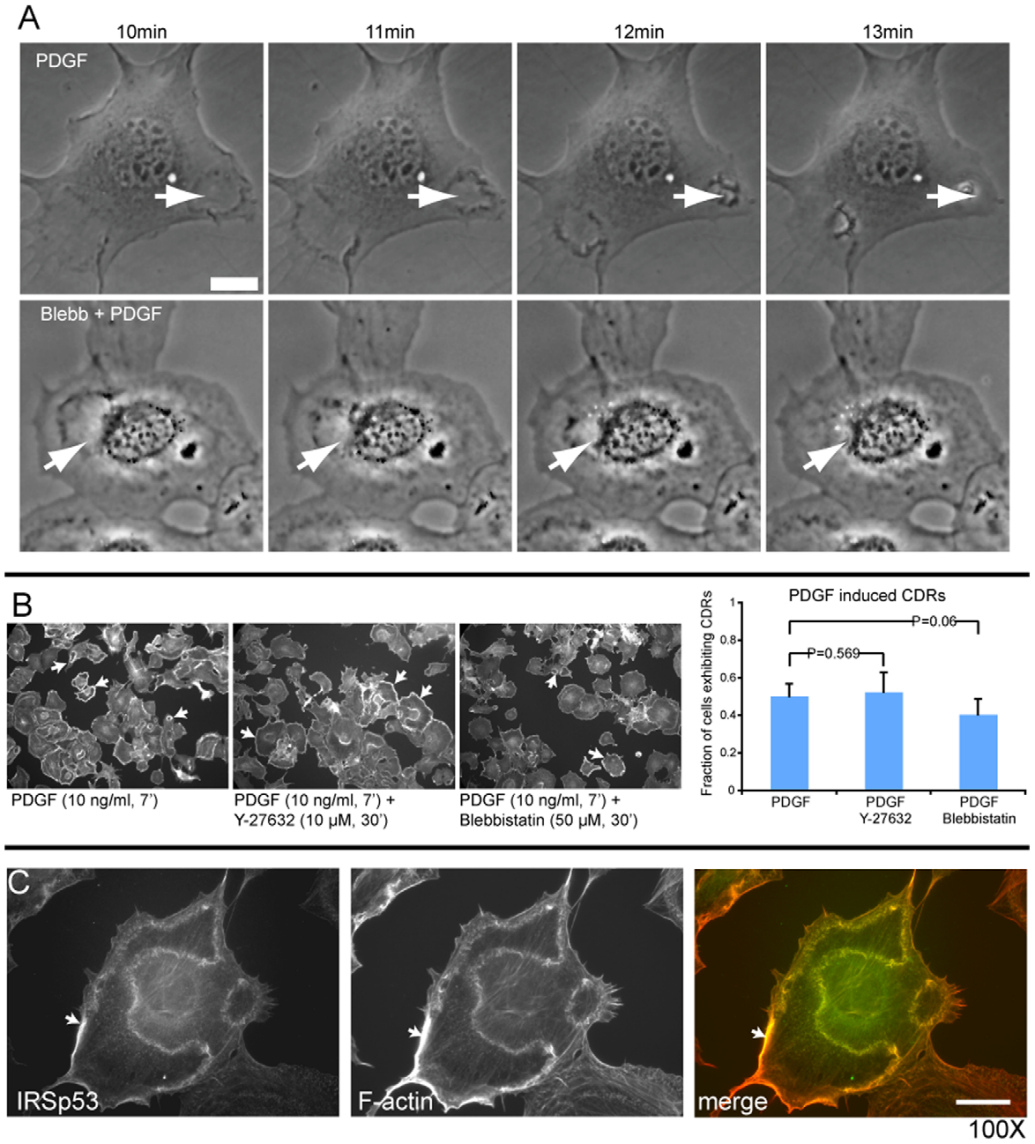
Experimental results. Experiments done in MEF cells which are stimulated by PDGF. (A) Time-lapse of CDRs dynamics. Still images of MEF cells serum-starved and pre-treated with vehicle (upper panels) or Blebbistatin (lower panels) and subsequently treated with PDGF to induce CDRs formation. CDR dynamics were recorded by time-lapse video microscopy (see also Movie S1 and Methods section). Bar, 20 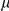
m. (B) The fraction of cells exhibiting CDRs is unaffected by treatment with two different myosin II inhibitors. P-values show no statistical significance. (C) IRSp53 is localized at CDRs. IRSp53 marked in green and actin in red. Bar 10 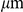. Arrows denotes CDRs.

In this paper, we are particularly interested in the phenomenon of Circular Dorsal Ruffles (CDR), which form on the apical surface of cells as circular actin rings that eventually enclose, generating an endocytic vesicle [4] (Fig. 1). These CDRs are involved in internalization of the membrane and its receptors, and are induced by ligand stimulation of membrane receptors, mainly of the tyrosine kinase family. These dynamic structures are driven by actin polymerization, which is initiated by membrane bound activators, such as N-WASP and WAVE complex [4], [11]. CDRs are formed in response to excitation of the cell by growth factor.

In order to test whether CDRs are dependent on actomyosin contractility, as suggested in [8], mouse embryo fibrobalsts were treated with two types of myosin II inhibitors (Y-27632 and Blebbistatin), and showed that CDRs are largely independent of actomyosin contractility (Fig. 1a,b). The observed velocities for CDRs in normal and blebbistatin-treated cells are 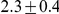 and 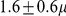
m/sec respectively. This difference in velocities is not statistically significant (see Movie S1).

There has been evidence that the actin activator N-WASP is recruited to CDRs by a curved membrane protein called Tuba [12]. Tuba is a protein that contains the Bin/Amphiphysin/Rvs (BAR) domain [13], which is known to bend membranes in a concave shape [14]. In addition, we present new experimental observations that indicate the localization in CDRs of IRSp53 protein (Fig. 1c), which contains the Missing–in–metastasis (MIM) domain, and induces convex membrane shape [15]. This protein was also shown to have the ability to recruit actin activating proteins [16].

### Theoretical Results

**Figure 2:**
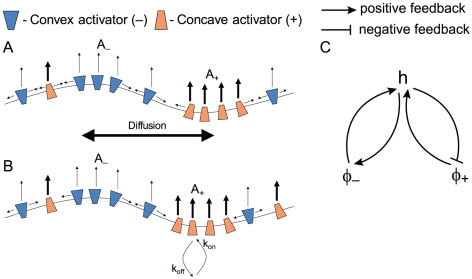
Schematic description of the model. (a) The activator diffuses in the membrane. (b) The activator adsorbs to the membrane from an infinite reservoir. (c) Feedback diagram describing the main interactions in our model, where positive and negative feedback loops combine to produce oscillations.

Motivated by these observations, we propose here a model for CDRs, which is based on the interplay between two types of protein complexes that contain an activator of actin polymerization and a curved membrane protein; one type is convex while the other is concave in shape (Fig. 2). For example, one such concave complex may contain Tuba and N-WASP [12], and a convex complex may contain IRSp53 and WAVE [16]. Note that we explore here the minimal model that contains just one type of activator of each type of curvature (concave and convex), while in the real cell many different proteins of both curvatures coexist and may play a role in CDR formation, as we indicate in Text S1.

In our model we include the following three components (Fig. 2): the flexible cell membrane, and the concentration fields of the membrane-bound activators of the two types of curvatures. The membrane has the usual bending and stretching elasticity, and is assumed to be flat when there are no activators present. The activators induce a spontaneous curvature on the membrane, proportional to their local concentration. The membrane is further pushed by actin polymerization, which is proportional to the local concentration of the activators. In turn, the dynamics of the activators is influenced by the membrane shape, causing the activators to aggregate where the local membrane shape more closely matches their spontaneous curvature. In the cell the activators both diffuse in the membrane and adsorb from the cytoplasm. In order to analyze the influence of the two processes separately and to keep the analysis simple, we will assume that each activator can be either diffusive or adsorptive but not both (Fig. 2a,b). We analyze all possible sets of different types of dynamics. This is a mean-field, continuum model, whereby we do not describe the small-scale shape of the membrane due to the individual activators, but treat only the averaged (coarse-grained) membrane shape.

The feedback mechanisms (Fig. 2c) that operate in our model, couple the distribution of the curved activators on the membrane to the membrane shape (curvature). The activators tend to localize where the membrane has a curvature that matches their spontaneous shape, while they in turn modify the membrane shape due to the forces that they apply; one force is simply due to their shape which tends to curve the membrane, and the other, active force is due to the recruitment of actin polymerization, and is purely protrusive. The convex activators alone can give rise to a positive feedback with the local membrane deformation, whereby they tend to form membrane protrusions in which they are highly localized [7], [17], but do not propagate laterally. The concave activators alone give rise to a negative feedback with the membrane deformation, resulting in damped oscillations [7]. Combining the two types of activators can give rise to unstable waves, whereby the convex activators initiate a protrusion, which is then modified by the aggregation of concave activators that tend to inhibit the local instability, but end up only shifting it laterally in space. This is how the propagating waves arise in our model from the interplay between the positive and negative feedbacks of the two curved activators and the membrane shape.

The membrane is characterized by height undulations 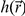, while the area coverage fractions of the convex and concave activators are denoted by 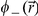 and 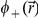. The proportionality factors relating the local concentration of activators to the protrusive actin force that they induce, are denoted by 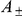 respectively. We will denote the activator dynamics by the dynamics of the convex followed by the dynamics of concave activator, e.g. diffusion(−)–adsorption(+). We are looking for the regimes of parameters where the system supports undamped propagating waves. We use linear stability analysis to map the regimes of parameters where the system becomes unstable, and complement this analysis with simulations that include the non-linearity due to conservation of the diffusive activators (Eq. 5). We find below that indeed the model we describe has regimes in which unstable waves arise, even in the limit of small perturbations (linear analysis).

**Figure 3:**
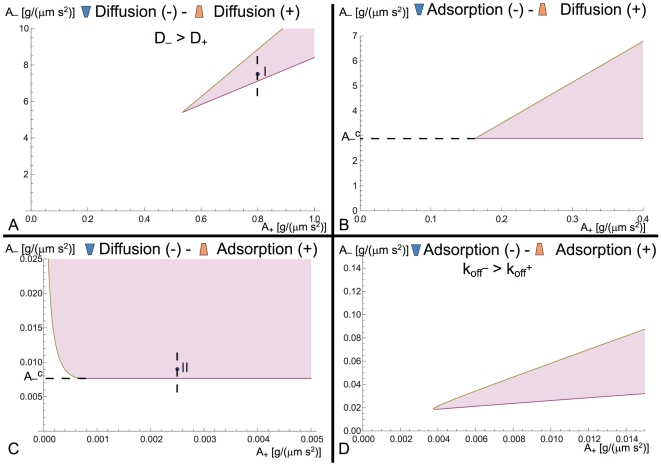
Wave instability phase diagram in the 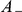
–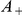 plane. Regions marked in pink denote the unstable waves. (a) the diffusion(−)–diffusion(+) model, when 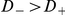. (b) the adsorption(−)–diffusion(+) model. (c) the diffusion(−)–adsorption(+) model. (d) the adsorption(−)–adsorption(+) model when 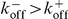. In (a) and (c) the dashed line marks the values along which the bifurcation graph (Fig. 5) was plotted. In (b) and (c) the threshold value of 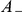 is denoted by 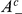.

We analyze the linear stability of the system as a function of the activity levels of the two activators, i.e. in the 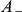
–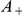 plane, in Fig. 3 (parameters used in these calculations are given in Table 1). We chose to analyze the system in terms of these parameters because cells can regulate the activity of the actin cytoskeleton through a variety of signaling pathways [4], and these are also experimentally accessible. In Fig. 3 we show only the regions of wave instability, and a more detailed analysis of these phase diagrams is given in Text S1. The following general conclusions can be drawn from the phase diagrams in Fig. 3

**Table 1:** List of parameters used in the calculations.

Parameter	Units	Value^a^	Parameter	Units	Value^a^
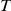	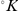	300	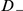 b	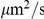	1.3, 1
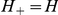	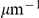	10	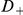 c	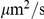	1
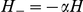	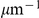	-1	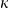	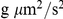	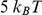
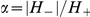	a.u.	0.1	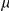 d,e	a.u.	-1
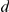	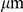	0.1	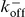 d	s 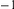	0.02, 0.01
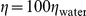	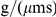	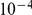	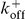 e	s 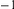	0.01
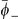 b	a.u.	0.5, 0.8	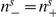	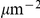	500
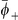 c	a.u.	0.5, 0.8	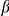	a.u.	10

a Dynamic constants were estimated from [32] and spontaneous curvatures from [16], [21]. Other values are of typical magnitude for cells.

b First number corresponds to diffusion(−)–diffusion(+) model and the second number corresponds to the diffusion(−)–adsorption(+) model.

c First number corresponds to diffusion(−)–diffusion(+) model and the second number corresponds to the adsorption(−)–diffusion(+) model.

d Relevant for adsorption(−)–diffusion(+) model.

e Relevant for diffusion(−)–adsorption(+) model.

1. When the dynamics of both activators is of the same type (both adsorptive or diffusive - a, d), we see that for unstable waves to arise the convex activator (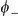) needs to have faster dynamics than the concave activator (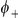). The convex activator is the one responsible for the instability in our system, as it has a positive-feedback with the membrane shape (Fig. 2), and it therefore needs to respond faster to the membrane deformations, as compared to the concave activators which have a negative feedback with the membrane shape.

2. In all the cases we find that unstable waves occur above some minimal value of both 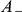 and 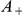. Note that for all the cases except the diffusion(−)–adsorption(+), the unstable waves disappear for 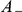 above some critical value (a,b,d).

3. When the activators have different types of dynamics (b, c) the transition from damped waves to unstable waves is given approximately by a constant threshold value of 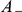, denoted by 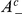 (red line). In both cases this critical value increases with increasing membrane tension. Only for case (c), we find that above a critical value of the membrane tension, unstable waves appear even for vanishing 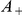.

**Figure 4:**
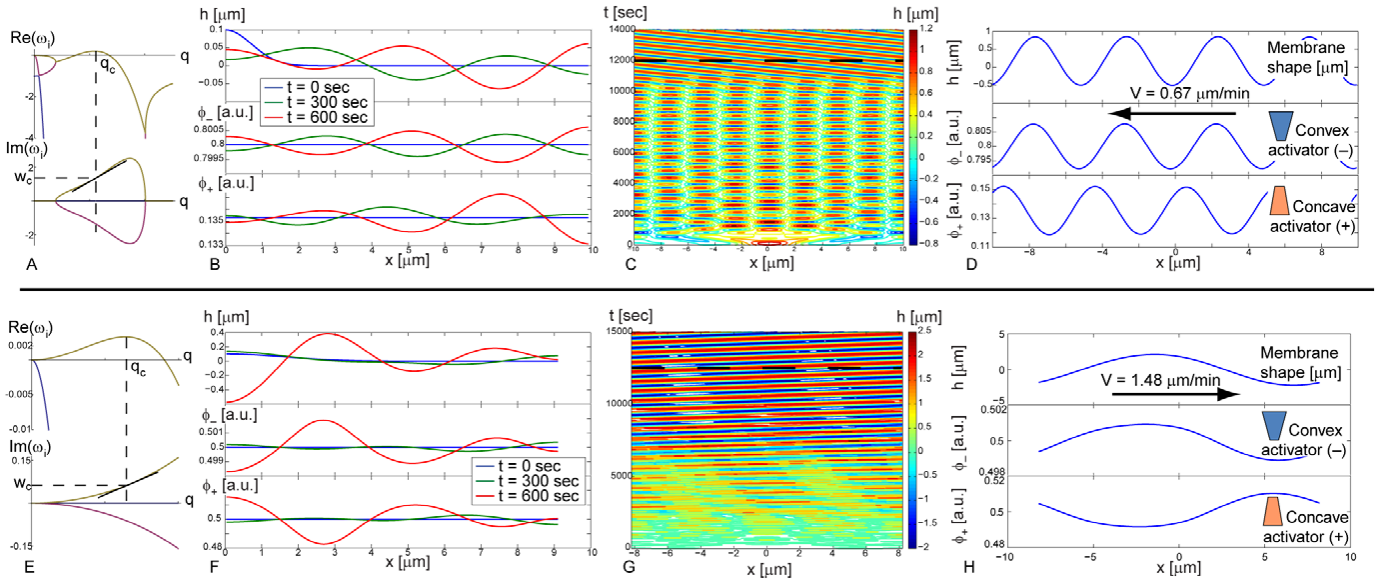
Linear stability and simulation results. (a–d) Results of the diffusion(−)–adsorption(+) system. (a) Dispersion relation of point marked II in Fig. 3c. Vertical dashed line mark 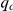 and horizontal dashed line marks 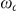. The slope of the imaginary part of the dispersion relation at 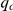 gives us an estimate of the group velocity of the waves 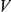. (b) Simulation for short times. One can see that the convex activators are in-phase with the membrane while the convex activators are in anti-phase. Due to symmetry only half of the domain is shown. (c) Kymograph depicting the membrane height displacement as a function of space and time. (d) Steady state wave at time t = 12,500 sec (marked by the dashed line in (c)). Arrow shows direction of propagation. (e–h) Results of the diffusion(−)–diffusion(+) system. (e) Dispersion relation of point marked I in Fig. 3a. Vertical dashed line marks 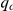 and horizontal dashed line marks 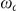. (f) Simulation for early times (as in (b)). (g) Kymograph depicting the membrane height displacement as a function of space and time. (h) Steady state wave at time t = 12,000 sec (marked by the dashed line (g)). Arrow shows direction of propagation. The simulations are shown in Movies S2 and S3 respectively.

We now explore in more details the cases of diffusive(−)–adsorptive(+) (a) and diffusive(−)–diffusive(+) (c) dynamics. In Fig. 4, we give the dynamics of the waves for parameter values that support unstable waves (points marked II and I in Fig. 3a,c respectively). We plot the dispersion relation and the time evolution simulation of the waves both for short times and at the final steady-state, from an initial small perturbation. In the dispersion relations the modes that support unstable waves are characterized by having a non-vanishing imaginary part, and a positive real part.

From the dispersion relation for the diffusive(−)–adsorptive(+) case (Fig. 4a) we find that the unstable waves exist for a limited range of wavelengths, around 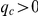. We show in Fig. 4b the result of a simulation for short times, where we find that the most dominant wavelength that propagates away from the initial perturbation is indeed 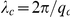, which has the largest positive real part in the dispersion relation and is therefore the most unstable mode (Fig. 4a). An approximate expression for 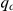 is given in Text S1. We find from this expression that the wavelength 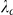 depends more strongly on the activity of the convex activator, as 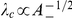. It depends very weakly on the activity of the concave activator 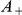.

A simulation for the long time evolution of the waves is shown in Fig. 4c (see Movies S2 and S3). We find that the initial perturbation induces counter-propagating waves and therefore a standing-wave pattern fills the domain, at the most unstable wavelength 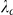, with an oscillation period which is close to that predicted by the linear dispersion relation (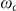 in Fig. 4a). Eventually, numerical noise breaks the symmetry of the counter-propagating waves, and a single traveling wave persists at wavelength 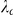 (Fig. 4d). The time it takes the system to break the symmetry is determined by noise, which is not included explicitly in these calculations. The velocity of this wave is 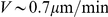, which is smaller by about 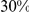 compared to the group velocity predicted by the slope of the dispersion relation at 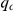 (Fig. 4a). A good approximation for the wave velocity is given by
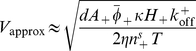
(1)See Materials and Methods section for the definition of the different parameters and the derivation of this expression. As is shown in Eq. 1, the velocity increases with the strength of the active forces (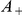), and the rate of activator turnover (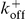), as well as with the membrane bending modulus (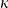). The velocity decreases for increasing fluid viscosity (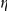). From this approximation we understand that the velocity depends very weakly on the activity of the convex activators (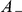). The accuracy of this approximate expression is discussed below.

In Fig. 4e–h we plot the analysis of the diffusive(−)–diffusive(+) system. The main difference in this system is that the unstable waves extend to infinite wavelengths (Fig. 4e). At short times (Fig. 4f) the most unstable wavelength (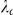) dominates, but non-linear interactions eventually cause the largest wavelength possible in the domain to persist (Fig. 4g,h). The velocity of this wave is 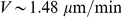, which is smaller by about 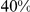 compared to the group velocity predicted by the slope of the dispersion relation at the wavelength of steady-state wave.

In both cases we find that in the propagating waves the convex activator (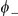) is in-phase with the membrane displacement, while the concave activator (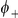) is almost in anti-phase (Fig. 4d,h).

**Figure 5:**
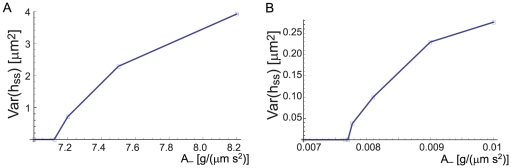
Bifurcation analysis. The mean square amplitude of the membrane height displacement in the two systems: (a) diffusion(−)–diffusion(+), (b) diffusion(−)–adsorption(+), along the vertical dashed lines in Fig. 3a,c respectively. The amplitude of the steady-state waves continuously vanishes as we approach the wave instability transition line from above (supercritical bifurcation).

In Fig. 5 we plot the mean-square amplitude of the steady-state membrane waves as a function of the activity of the convex activators, moving along the vertical dashed lines in Figs. 3a,c. We find that the amplitude of the steady-state waves continuously vanishes as we approach the wave instability transition line (red lines in Figs. 3a,c) from above (supercritical bifurcation).

## Discussion

Experimental evidence given here demonstrates that CDRs contain curved membrane proteins of both curvatures which are furthermore known to be involved in the recruitment of actin polymerization to the membrane. In addition, myosin II contractility was shown not to be an essential component of such waves, and its inhibition does not change the wave velocity. Our theoretical model demonstrates that indeed actin protrusive forces induced by the interplay of these two types of membrane-bound curved activators is sufficient to give rise to propagating membrane waves (Fig. 2c). Therefore this result suggests that this could be the dominant mechanism for CDRs.

We can make the following more quantitative comparisons between the waves that our model gives and the observed CDRs.

1. For the cases where the concave activator is adsorptive, the waves in our model have a typical wavelength of order of a few microns (for “rule of thumb” parameter values, Table 1), which is similar to the width of observed CDRs [11], [12].

2. The experimentally observed wave velocity is in reasonable agreement with the the range of velocities we observe in our model.

3. The concave and convex activators are displaced within the propagating CDR, such that the convex activator is localized at the membrane protrusion, while the concave activators are localized where the membrane is depressed (Fig. 4b,f). This may explain the observation that Tuba trails the actin front in CDRs [12].

These comparisons support the validity of our model for CDR, and may further indicate that the concave complex (e.g. containing Tuba) is more slowly diffusing in the membrane compared to the convex complex (e.g. containing IRSp53).

Regarding the velocity of the waves in our model, Eq. 1 shows that it depends on both the passive parameters of the system (such as the membrane elasticity and fluid viscosity) and on the average concentration and activity of the concave activators (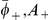). This expression highlights that the wave phenomenon that we describe is a result from an interplay between the active forces due to actin polymerization and the passive reaction of the system. Note that the approximate expression we derived for the wave velocity (Eq. 1) is reminiscent of the expression that appears for myosin-II driven membrane waves (Eq. 5 in [8]).

Our model gives the following insight about the process of CDR excitation in cells. Before the cell is excited its internal parameters correspond to a point in the stable regime of the phase diagram (below the red line in Fig. 3). When it is excited the stimulation changes the internal parameters, for example the activity of the actin activators (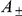), and above some threshold values the system crosses into the unstable-wave region. An alternative possibility could have been that the cell can be close enough to the transition line (in the stable regime), such that a large perturbation switches it to the propagating wave state. This route does not exist within our non-linear model, as illustrated in Fig. 5. This means that the difference between a quiescent cell and an excited cell with CDRs is a real change in the internal state of the cytoskeleton activity, and not simply a large perturbation of the membrane-cytoskeleton organization.

Let us discuss some assumptions that we have used in our model. We assumed that the actin polymerization induced by the curved activators (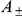) is spatially uniform. However, there are mechanisms in the cell that can make this parameter vary in space since it may depend on the local membrane curvature [18] and signaling pathways [19]. Our model demonstrates that even without this added level of complexity propagating waves can form. Furthermore, our simulations were done in a regime where the amplitude of the concentration undulations of the activators in the waves are small (Fig. 4d,h), and as a result the waves are purely periodic in space. In comparison, the observed CDRs are solitary (Fig. 1) and the actin activators are highly localized in the CDR. Nevertheless, the conditions that allow the system to support waves are independent of the amplitude of the wave (Fig. 5), so our conclusions remain unaffected. As soon as we reduce the membrane tension and allow the membrane amplitude to form stronger gradients, we got complete depletion of activators from certain regions of the membrane, and this indicates that the system has then the tendency to form isolated structures, similar to the solitary waves observed experimentally. A simulation of a solitary propagating structure, which shows that such structures indeed tend to form in our model, is shown in Movie S5. This regime remains to be explored in future studies.

**Figure 6:**
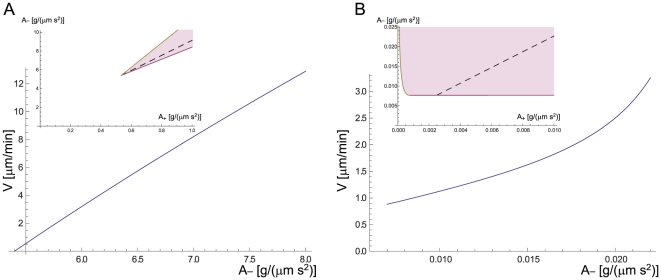
Group velocity dependence on 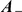. The group velocity dependence along the dashed lines in the insets: (a) the diffusion(−)–diffusion(+) model, when 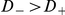. (b) the adsorption(−)–diffusion(+) model. This trajectory represents the effects of addition of actin polymerization inhibitors or promoters. In both cases we find that the wave velocity increases with the actin activity, but in a very different manner. This prediction can serve to differentiate between the different types of activator dynamics described by our model.

The different versions of our model (Fig. 3) give different behavior for the propagating waves, as can be seen in the final wavelengths in Fig. 4. Future experiments may allow to distinguish between the different versions of our model. One example for such a discriminating observation between the models is shown in Fig. 6, where we plot the calculated dependence of the wave group velocity on the actin polymerization activity. This actin activity may be modified experimentally by using a variety of actin inhibitors or promoters, which would therefore change both 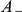 and 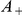. The plotted trajectory is schematic, as it assumes a simple linear relation between the response of both types of activators to the drug.

**Figure 7:**
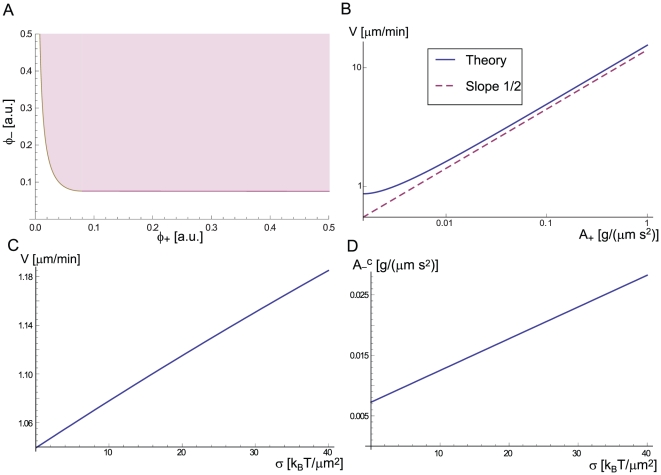
Predictions for the diffusion(−)–adsorption(+) model. (a) Wave instability phase diagram in the 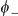
–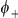 plane. It is very similar to the phase diagram in the 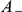
–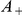 plane (Fig. 3c). (b) Log-log plot of the dependence of the group velocity at 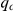 (Fig. 4a) on the parameter 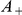, along the wave instability transition line in Fig. 3c. The dashed line gives the approximate expression for the velocity, given in Eq. 1. (c) The dependence of the group velocity at 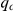 (for 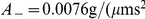), 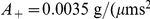)), on the membrane tension. (d) The dependence of the threshold value 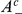 (Fig. 3c) on the membrane tension.

We can use our model to make the following list of observable predictions: (i) functional or genetic interference with one type of curved proteins (assuming non-redundant roles among proteins of the same type of curvature, see Text S1 section 2) should inhibit CDR formation, (ii) the two types of curved activator complexes are spatially displaced within the CDR, following the undulation in the membrane shape (Fig. 4b,f), (iii) the phase diagrams shown in Fig. 3 may be explored systematically by controlling the rate of actin polymerization in the cell (note that drugs such as Latrunculin A would change both 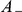 and 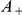, Fig. 6), (iv) the expression levels of the two types of activators may be regulated artificially and would change the behavior of the cell (shown in Fig. 7a), (v) the CDR velocity should increase roughly as a square-root of the activity of the concave activator, 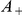 (Eq. 1, Fig. 7b), and (vi) change of the membrane tension will change the velocity of the CDR and the threshold value of 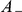 for wave instability (Figs. 7c,d respectively).

In Fig. 7b the accuracy of the approximate expression for the wave group velocity given in Eq. 1 can be judged, as a function of 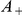, by comparing to the group velocity at 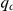.

We present a physical model that demonstrates how actin protrusive forces induced by the interplay of membrane-bound curved activators of both convex and concave curvatures, can give rise to propagating membrane waves. This is a new mechanism for membrane-cytoskeleton waves, and may be the dominant driving force for CDRs. Our model explains many of the observed features of CDRs and provides testable experimental predictions. The theoretical model, together with the experimental observations, demonstrate the essential role played by curved membrane proteins that recruit actin polymerization as organizers of the cortical actin cytoskeleton. Unlike other cellular structures that have been shown to contain such proteins [20], [21], we demonstrate that proteins of both curvatures are necessary to drive propagating waves.

## Materials and Methods

### Drug treatment and staining

In order to test whether CDR induced by PDGF stimulation are dependent on an intact actomyosin contractile system, mouse embryo fibroblasts (MEF) were serum-starved and pre-treated with vehicle or Y-27632 (10 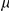
M, 30′), a specific inhibitor of ROCK kinase, that regulates myosin light chain kinase and MLC-based contractility [22], or Blebbistatin (50 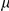
M, 30′), a small molecule inhibitor showing high affinity and selectivity toward myosin II [23] (Fig. 1a,b). Cells were subsequently treated with 10 ng/ml of PDGF for 7 min, which potently and synchronously induces CDR fomation [24] in MEFs. Cells were then fixed and stained with rhodamine-phalloidin to detect F-actin and visualize CDR. The percentage of MEFs exhibiting CDRs were counted. Data is expressed as mean 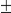 SD (Fig. 1b). To detect the localization of IRSp53 in CDRs, cells were fixed and stained with anti-IRSp53 antibody (green) and rhodamine-phalloidin to detect F-actin (red)(Fig. 1c).

### Cell culture and reagents

Mouse embryo fibroblasts (MEFs) used in the experiments were derived as described in [25] from Eps8 null mice. MEFs were cultured in DMEM-Glutamax-1 medium supplemented with 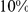 FBS, 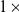
Pen-Strep. IRSp53 knockout cells were spontaneously immortalized cells from IRSp53 knockout mouse embryos infected either with pBABE-puro or pBABE-puro-IRSp53 [26]. MEFs were cultured in DMEM-Glutamax-1 medium supplemented with 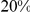 FBS, 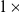
Pen-Strep, and 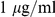 puromycin. The monoclonal anti-IRSp53 was generated against the full-length His-tagged purified protein [27]. PDGF was from Immunological Science (Rome, Italy), Blebbistatin from Sigma-Aldrich (St. Louis, MO, USA), Y-27632 from Tocris Bioscience (Ellisville, MO, USA).

### Immunofluorescence microscopy and CDRs counting

Cells seeded on gelatin were serum starved for two hours and then treated with PDGF for 7 minutes. Cells were then processed for indirect immunofluorescence microscopy. Briefly, cells were fixed in 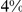 paraformaldehyde for 10 min, permeabilized in 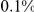 Triton X-100 and 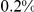 BSA for 10 min, and then incubated with the primary antibody for 45 min, followed by incubation with the secondary antibody for 30 min. F-actin was detected by staining with rhodamine-phalloidin (Sigma-Aldrich, St. Louis, MO, USA) at a concentration of 6.7 U ml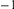. The number of cells exhibiting CDRs upon PDGF treatment was counted. At least 500 cells in each experiment performed in triplicate were analyzed (mean s.e.m.).

### Time lapse of CDRs

MEFs cells seeded on gelatin were serum-starved for two hours and then pre-treated with vehicle or Blebbistatin. Cells were treated with PDGF and subjected to time-lapse video microscopy at 37°C, 5% CO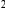 using an Olympus IX81 microscope (40X objective) connected to a Photometrics cascade 1K camera. Images were taken every 5 seconds for 20 minutes. Reduction of the area of each CDR was monitored over time using Image-J software and from the relation between the area and time we could extract the reduction in the average radius, by assuming a circular shape. We then used the change in the CDR radius at the beginning of the shrinking, to calculate the velocity.

### Model details

The membrane is characterized by height undulations 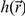 (Monge representation in the limit of small undulations), while the area coverage fractions of the convex and concave activators are 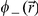 and 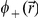, with spontaneous curvature 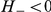 and 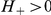. The dynamics are governed by the Helfrich Hamiltonian [28] where the bending energy is proportional to the mismatch between the mean membrane curvature (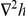) and the spontaneous curvature of the curved activators (up to quadratic order)
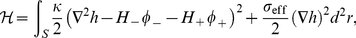
(2)where 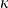 is the membrane's bending modulus and 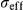 is an effective surface tension which includes contributions due to the spontaneous curvature and entropy of the activators (details in Text S1).

We assume that the pushing force of actin polymerization is linearly proportional to the activators' density
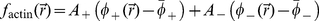
(3)where 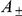 is a proportionality constant that gives a measure of the activity of the actin polymerization induced by the respective activator and 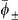 is the average concentration. We will assume in this work that the values of 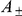 are uniform throughout the domain and constant in time. The 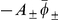 terms in Eq .3 are equivalent to a uniform displacement of the entire membrane (Galilean transformation) which does not change the shape evolution. We obtained similar results when the analysis was carried out using an osmotic pressure restoring force (see Text S1).

The elastic forces acting on the membrane are derived variationally from the free energy, which is the energy (Eq. 2) plus the entropy of the activators. Together with the forces due to actin polymerization (Eq. 3) we get
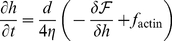
(4)assuming local hydrodynamic interactions, where 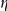 is the viscosity of the fluid surrounding the membrane and 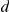 is the typical extent of the hydrodynamic interactions [8], [17], which represents the effective distance of fluid flow between the membrane and the cytoskeleton elements [29]. This approximation of local hydrodynamic interactions is more relevant for a membrane near a dense network of actin filaments, which is the situation for membranes that are deformed by the cortical actin cytoskeleton [30]. Note that Eq. 4 describes how the membrane shape is locally dependent on the activators' distribution which promote the actin protrusive force, leading to an increase in 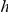 (feedback scheme Fig. 2c).

We consider two distinct cases for the dynamics of the activators, either diffusive in the membrane or adsorptive from the cytoplasm. For the case of diffusive dynamics the total amount of activators is conserved, so the equation of motion derived from the free energy (details in Text S1) is given by
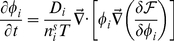
(5)where 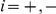, 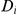 is the diffusion coefficient of the curved activator, 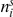 is the saturation concentration i.e. the maximal concentration at which these complexes cover the whole cell membrane and 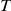 is the temperature. Note that the current of activators in response to the local membrane curvature, is proportional to: 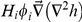. This term in Eq. 5 describes how the diffusive activators' distribution depends on the local membrane shape (curvature), since this current of activators carries them towards regions where the membrane curvature matches their spontaneous shape (feedback scheme Fig. 2c).

For the case of adsorptive dynamics, the rate constants of the binding/unbinding process are governed by a Boltzmann factor of the mismatch in the bending energy between the local membrane curvature and the activator's spontaneous curvature
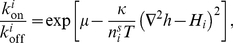
(6)where 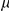 is the chemical potential describing the affinity for adsorption on a membrane of matching curvature, and the equation of motion for 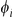 is of first-order kinetics in the form
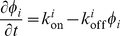
(7)where we assume that the cytoplasmic concentration of curved activators is approximately constant and uniform due to the fast diffusion of proteins in the cytoplasm, compared to the typical oscillation time of the waves. For small undulations of the membrane, the equation is linear in the curvature 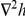. Eq. 7 describes how the adsorptive activators' distribution depends on the local membrane shape (curvature), since they adsorb in regions where the membrane curvature matches their spontaneous shape (feedback scheme Fig. 2c).

### Linear stability analysis

For the linear stability analysis we linearize the equations of motion, for all types of dynamics (Eqs. 4, 5, 7). We expand in small deflections around the uniform steady-state, where the membrane is flat and the uniform concentrations are 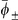. The domain of wave instability is bounded by the red and brown lines in Fig. 3 (calculated in Text S1). In this region there are oscillatory unstable modes where: 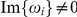 and 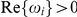. The amplitude of these modes grow exponentially from small initial perturbations, and oscillate or propagate on the membrane surface. The system is stable below the red line, such that initial perturbations decay exponentially: 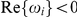.

### Non-linear simulations

The one-dimensional simulations are done using a finite-difference scheme for the full nonlinear model with translational symmetry, using Matlab. We used periodic boundary conditions, and the initial perturbation in the membrane shape was Gaussian (uniform initial distributions of the activators). The exponential growth in the amplitude of the membrane wave is arrested in the real cell due to the finite membrane area, which we describe by adding a non-linear tension term [31], in the form: 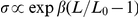, where 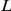 is the total membrane length, 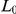 is the initial length and 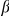 is the non-linear coefficient. We used a value of 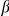 which limited the amplitude of the waves to be of order 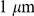, as is estimated for CDRs.

### Strong concave activator approximation

In the diffusive(−)–adsorptive(+) model, for strong concave activator levels (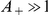) we can gain a deeper understanding of the source of the wave velocity. In this limit we can simplify Eqs. 4, 7, neglecting the effect of the forces due to the convex activator, and get
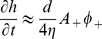
(8)

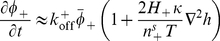
(9)From these equations we can derive a wave equation of the form
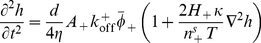
(10)with the wave velocity given in Eq. 1. In this limit the dispersion relation is acoustic-like, it is almost linear in 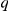.

## Supporting Information

Text S1 PDF of the supporting information file.
(PDF)

Movie S1
**Time-lapse video microscopy of MEF cells serum-starved and pre-treated with vehicle (upper panels) or Blebbistatin (lower panels) and subsequently treated with PDGF to induce CDRs fomation (see Methods section for details).** The film segment shown starts 10 minutes after PDGF addition and lasts 3.5 minutes. Bar, 20 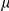
m.(AVI)

Movie S2 **Simulation for the diffusion(-)–adsorption(+) system (** **Fig. 4b–d** **).** The top panel shows the membrane height displacement, the middle panel gives the concentration distribution of the convex activator, and the bottom panel gives the concentration distribution of the concave activator.
(AVI)

Movie S3 **Simulation for the diffusion(-)–diffusion(+) system (** **Fig. 4f–h** **).** The top panel shows the membrane height displacement, the middle panel gives the concentration distribution of the convex activator, and the bottom panel gives the concentration distribution of the concave activator.
(AVI)

Movie S4 **Simulation for the formation and coalescence of protrusions in the diffusion(-)–diffusion(+) system.** The top panel shows the membrane height displacement, the middle panel gives the concentration distribution of the convex activator, and the bottom panel gives the concentration distribution of the concave activator.
(AVI)

Movie S5
**Simulation of solitary propagating structure that arose within our model when we removed the effects of the non-linear tension (** 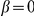 **), using the same values for the parameters as used for the calculation shown in** **Fig. 4B–D** **(diffusion(-)–adsorption(+).** In this calculation we used the full expression for the exponential form of the adsorption of 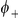 given in Eq.S6. Note that the membrane amplitude is clearly beyond the validity of the Monge representation of the membrane curvature. In this simulation we find that the CDR has a central bump where the convex activators are localized, while the concave activators form displaced bands at the front and the back.(AVI)
