# Supplementary material for: Propagating Cell-Membrane Waves Driven by Curved Activators of Actin Polymerization
Source: PLoS One. 2011 Apr 21;6(4):e18635. doi: 10.1371/journal.pone.0018635 (PMC3080874; doi:10.1371/journal.pone.0018635)
Supplement: Text S1 — PDF of the supporting information file. (PDF) [file pone.0018635.s001.pdf]

Supplementary Information
Propagating cell-membrane waves driven by curved activators of actin polymerization

Barak Peleg, Andrea Disanza, Giorgio Scita and Nir S. Gov

March 28, 2011

Contents

1 Role of IRSp53 in CDR formation **2**

2 Mathematical derivation **2**
2.1 Derivation of free energy . . . 2
2.2 Derivation of equations of motion . . . 3
2.2.1 Membrane . . . 3
2.2.2 Diffusive activator . . . 3
2.2.3 Adsorptive activator . . . 3
2.3 Transformation to dimensionless form . . . 3
2.3.1 Diffusion(-) – diffusion(+) model . . . 3
2.3.2 Other models . . . 4
2.4 Linear stability analysis . . . 4
2.4.1 Diffusion(-) – diffusion(+) model . . . 4
2.4.2 Other models . . . 5

3 Explanation of transition lines **5**
3.1 Turing instability transition . . . 5
3.1.1 Turing instability from a stable mode . . . 5
3.1.2 Turing instability from a unstable wave mode . . . 6
3.2 Wave transition . . . 6
3.3 Wave instability transition . . . 6
3.3.1 Far away from a Turing instability region . . . 6
3.3.2 Adjacent to a Turing instability region . . . 6

4 Explanation of the phase diagrams in Fig. S2 **6**
4.1 Diffusion(-) – diffusion(+) model . . . 8
4.2 Adsorption(-) – adsorption(+) model . . . 8
4.3 Diffusion(-) – adsorption(+) model . . . 9
4.4 Adsorption(-)–diffusion(+) model . . . 9

5 Simplified diffusive(-)–adsorptive(+) model **10**

6 Examples of dispersion relations **10**

7 Method of holding the membrane **10**

8 Turing instability transition line in the diffusion(-)–adsorption(+) model **12**

9 Non-linear membrane tension **12**

10 Turing instability **12**

1 Role of IRSp53 in CDR formation

To test the role of IRSp53 in CDR formation, we performed experiments comparing MEF KO-pB cells with MEF KO-pB-IRSp53 cells. As shown in Fig. S1 CDRs formed in both type of cells, hence IRSp53 removal does not affect PDGF induced CDRs formation. This observation may indicate that

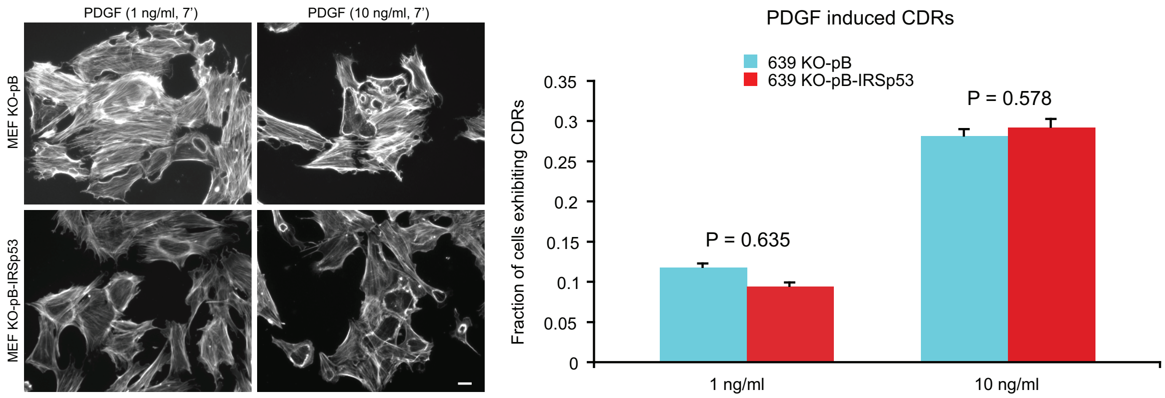

Figure S1: IRSp53 removal does not affect PDGF induced CDRs formation.

in CDRs there are additional convex proteins that play a similar role to IRSp53 and maintain the formation of CDRs.

2 Mathematical derivation

2.1 Derivation of free energy

The most general free energy taking into account a Helfrich Hamiltonian and entropy of the activators (in an ideal gas approximation) is

$$\mathcal{F} = \int_S \left[\frac{\kappa}{2} \left(\nabla^2 h - H_- \phi_- - H_+ \phi_+ \right)^2 + \sigma + T \sum_{i=\pm} n_i^s \phi_i (\log \phi_i - 1) \right] \sqrt{1 + (\nabla h)^2} d^2 r, \tag{S1}$$

where κ is the bending modulus of the membrane and σ is the surface tension. Under the small undulation limit $\nabla h \ll 1$ we expect a linear equation of motion for h. So we can simplify the free energy to contain only quadratic terms in h, its derivatives and in the concentrations $\phi_\pm$:

$$\mathcal{F} = \int_S \frac{\kappa}{2} \left(\nabla^2 h - H_- \phi_- - H_+ \phi_+ \right)^2 + \frac{\sigma_{\text{eff}}}{2} (\nabla h)^2 + \left[T \sum_{i=\pm} n_i^s \phi_i (\log \phi_i - 1) \right] d^2 r, \tag{S2}$$

where σ_{eff} is defined as

$$\sigma_{\text{eff}} \equiv \sigma + \frac{\kappa}{2} \left(H_- \bar{\phi_-} + H_+ \bar{\phi_+} \right)^2 + T \sum_{i=\pm} n_i^s \bar{\phi_i} \left(\log \bar{\phi_i} - 1 \right) \tag{S3}$$

In our numerical calculations we took $\sigma_{\text{eff}} = \frac{\kappa}{2} \left(H_- \bar{\phi_-} + H_+ \bar{\phi_+} \right)^2$ because we could arbitrarily choose the value of σ to equal the surface tension contribution due to entropy of the proteins.

2.2 Derivation of equations of motion

The hydrodynamic interactions are introduced using the Oseen tensor for Stokes flow. The Oseen tensor, denoted here by $\mathcal{O}(\vec{r}) = d/(4\eta)\delta(\vec{r})$ according to [1] will take into account only local interactions due the confinement of the water molecules by the dense cortical actin mesh.

2.2.1 Membrane

$$
\begin{aligned}
\frac{\partial h}{\partial t} &= \int_S \mathcal{O}(\vec{r}-\vec{r}') \left[-\frac{\delta \mathcal{F}}{\delta h} + \sum_{i=\pm} A_i \left(\phi_i - \bar{\phi}_i\right) \right] d^2 r' \\
&= d/4\eta \left[-\kappa \nabla^4 h + \sigma_{\text{eff}} \nabla^2 h + H_- \kappa \nabla^2 \phi_- + H_+ \kappa \nabla^2 \phi_+ + A_-(\phi_- - \bar{\phi_-}) + A_+(\phi_+ - \bar{\phi_+}) \right] \quad \text{(S4)}
\end{aligned}
$$

2.2.2 Diffusive activator

$$
\begin{aligned}
\frac{\partial \phi_i}{\partial t} &= \frac{D_i}{n_i^s T} \nabla \left[\phi_i \nabla \left(\frac{\delta \mathcal{F}}{\delta \phi_i} \right) \right] \\
&= D_i \nabla^2 \phi_i + \frac{D_i H_i \kappa}{n_i^s T} \nabla \left[\phi_i \left(-\nabla^3 h + H_- \nabla \phi_- + H_+ \nabla \phi_+ \right) \right] \quad \text{(S5)}
\end{aligned}
$$

2.2.3 Adsorptive activator

In the adsorptive model the on and off rates are governed by a Boltzmann factor:

$$
\begin{aligned}
\frac{k_{\text{on}}}{k_{\text{off}}} &= \exp \left[\mu - \frac{\kappa}{n_i^s T} \left(\nabla^2 h - H_i \right)^2 \right] \\
&\simeq \left[1 - \frac{\kappa}{n_i^s T} \left((\nabla^2 h)^2 - 2 H_i \nabla^2 h \right) \right] \exp \left(\mu - \frac{\kappa H_i^2}{n_i^s T} \right) \\
&\simeq \left(1 + \frac{2 H_i \kappa}{n_i^s T} \nabla^2 h \right) \exp \left(\mu - \frac{\kappa H_i^2}{n_i^s T} \right) \quad \text{(S6)}
\end{aligned}
$$

and the equation of motion is

$$
\begin{aligned}
\frac{\partial \phi_i}{\partial t} &= k_{\text{on}} - k_{\text{off}} \phi_i \quad \text{(S7)} \\
&= k_{\text{off}} \left(1 + \frac{2 H_i \kappa}{n_i^s T} \nabla^2 h \right) \exp \left(\mu - \frac{\kappa H_i^2}{n_i^s T} \right) - k_{\text{off}} \phi_i
\end{aligned}
$$

2.3 Transformation to dimensionless form

From now on we will define the constant $\mathcal{O} \equiv d/(4\eta)$.

2.3.1 Diffusion(-) – diffusion(+) model

Transform the equations into dimensionless form using the following transformation:

$$
x \to \tilde{x} \sqrt{\frac{\mathcal{O} n_+^s T}{D_+ H_+}}, \quad t \to \tilde{t} \frac{\mathcal{O}}{\kappa} \left(\frac{n_+^s T}{D_+ H_+} \right)^2, \quad h \to \tilde{h} \frac{\mathcal{O} n_+^s T}{D_+} \quad \text{(S8)}
$$

and drop the tildes.

$$\begin{aligned}
\frac{\partial h}{\partial t} &= -\nabla^4 h + \frac{\sigma_{\mathrm{eff}}\mathcal{O} n_+^s T}{\kappa D_+ H_+^2}\nabla^2 h + \frac{H_-}{H_+}\nabla^2\phi_- + \nabla^2\phi_+ + \frac{A_-\mathcal{O} n_+^s T}{D_+ H_+^3 \kappa}(\phi_- - \bar{\phi}_-) + \frac{A_+\mathcal{O} n_+^s T}{D_+ H_+^3 \kappa}(\phi_+ - \bar{\phi}_+) \\
\frac{\partial \phi_-}{\partial t} &= \frac{D_- n_+^s T}{D_+ H_+^2 \kappa}\nabla^2\phi_- + \frac{D_- H_- n_+^s}{D_+ H_+ n_-^s}\nabla\left[\phi_-\left(-\nabla^3 h + \frac{H_-}{H_+}\nabla\phi_- + \nabla\phi_+\right)\right] \\
\frac{\partial \phi_+}{\partial t} &= \frac{n_+^s T}{H_+^2 \kappa}\nabla^2\phi_+ + \nabla\left[\phi_+\left(-\nabla^3 h + \frac{H_-}{H_+}\nabla\phi_- + \nabla\phi_+\right)\right] \qquad \text{(S9)}
\end{aligned}$$

2.3.2 Other models

The same can be done for the other models.

Adsorption(-) – diffusion(+) model

$$x \to \tilde{x}\left(\frac{\mathcal{O}\kappa}{k_{\mathrm{off}}}\right)^{\frac{1}{4}}, \quad t \to \tilde{t}/k_{\mathrm{off}}, \quad h \to \tilde{h} H_+ \sqrt{\frac{\mathcal{O}\kappa}{k_{\mathrm{off}}}} \qquad \text{(S10)}$$

Diffusion(-) – adsorption(+) model

$$x \to \tilde{x}\left(\frac{\mathcal{O}\kappa}{k_{\mathrm{off}}}\right)^{\frac{1}{4}}, \quad t \to \tilde{t}/k_{\mathrm{off}}, \quad h \to \tilde{h} H_+ \sqrt{\frac{\mathcal{O}\kappa}{k_{\mathrm{off}}}} \qquad \text{(S11)}$$

Adsorption(-) – adsorption(+) model

$$x \to \tilde{x}\left(\frac{\mathcal{O}\kappa}{k_{\mathrm{off}}^2}\right)^{\frac{1}{4}}, \quad t \to \tilde{t}/k_{\mathrm{off}}^2, \quad h \to \tilde{h}\frac{n_+^s T}{H_+}\sqrt{\frac{\mathcal{O}}{\kappa k_{\mathrm{off}}^2}} \qquad \text{(S12)}$$

2.4 Linear stability analysis

2.4.1 Diffusion(-) – diffusion(+) model

The homogenous fixed point is

$$\bar{h} = 0, \quad \bar{\phi}_-, \quad \bar{\phi}_+ \qquad \text{(S13)}$$

and the linearized equations around it are

$$\begin{aligned}
\frac{\partial h}{\partial t} &= -\nabla^4 h + \sigma_{\mathrm{eff}}\frac{\mathcal{O} n_+^s T}{\kappa D_+ H_+^2}\nabla^2 h + \frac{H_-}{H_+}\nabla^2\phi_- + \nabla^2\phi_+ + \frac{A_-\mathcal{O} n_+^s T}{D_+ H_+^3 \kappa}\phi_- + \frac{A_+\mathcal{O} n_+^s T}{D_+ H_+^3 \kappa}\phi_+ \\
\frac{\partial \phi_-}{\partial t} &= \frac{D_- n_+^s T}{D_+ H_+^2 \kappa}\nabla^2\phi_- + \frac{D_- H_- n_+^s \bar{\phi}_-}{D_+ H_+ n_-^s}\left(-\nabla^4 h + \frac{H_-}{H_+}\nabla^2\phi_- + \nabla^2\phi_+\right) \\
\frac{\partial \phi_+}{\partial t} &= \frac{n_+^s T}{H_+^2 \kappa}\nabla^2\phi_+ + \bar{\phi}_+\left(-\nabla^4 h + \frac{H_-}{H_+}\nabla^2\phi_- + \nabla^2\phi_+\right) \qquad \text{(S14)}
\end{aligned}$$

Fourier transforming the equations we get the stability matrix L,

$$\begin{pmatrix} \dot{h} \\ \dot{\phi}_- \\ \dot{\phi}_+ \end{pmatrix} = \begin{pmatrix} -q^4 - \frac{\sigma_{\mathrm{eff}}\mathcal{O} n_+^s T}{\kappa D_+ H_+^2} q^2 & -\frac{H_-}{H_+}q^2 + \frac{A_-\mathcal{O} n_+^s T}{D_+ H_+^3 \kappa} & -q^2 + \frac{A_+\mathcal{O} n_+^s T}{D_+ H_+^3 \kappa} \\ -\frac{D_- H_- n_+^s \bar{\phi}_-}{D_+ H_+ n_-^s} q^4 & -\left(\frac{D_- n_+^s T}{D_+ H_+^2 \kappa} + \frac{D_- H_-^2 n_+^s \bar{\phi}_-}{D_+ H_+^2 n_-^s}\right) q^2 & -\frac{D_- H_- n_+^s \bar{\phi}_-}{D_+ H_+ n_-^s} q^2 \\ -\bar{\phi}_+ q^4 & -\frac{H_- \bar{\phi}_+}{H_+} q^2 & -\left(\frac{n_+^s T}{H_+^2 \kappa} + \bar{\phi}_+\right) q^2 \end{pmatrix} \begin{pmatrix} h \\ \phi_- \\ \phi_+ \end{pmatrix} \qquad \text{(S15)}$$

2.4.2 Other models

Adsorption(-) – diffusion(+) model The homogenous fixed point is

$$\bar{h} = 0, \quad \bar{\phi}_- = \exp\left(\mu - \frac{\kappa H_-^2}{n_-^s T}\right), \quad \bar{\phi}_+ \tag{S16}$$

Diffusion(-) – adsorption(+) model The homogenous fixed point is

$$\bar{h} = 0, \quad \bar{\phi}_-, \quad \bar{\phi}_+ = \exp\left(\mu - \frac{\kappa H_+^2}{n_+^s T}\right) \tag{S17}$$

Adsorption(-) – adsorption(+) model The homogenous fixed point is

$$\bar{h} = 0, \quad \bar{\phi}_- = \exp\left(\mu - \frac{\kappa H_-^2}{n_-^s T}\right), \quad \bar{\phi}_+ = \exp\left(\mu - \frac{\kappa H_+^2}{n_+^s T}\right) \tag{S18}$$

From this the stability matrices can be derived in the same manner.

3 Explanation of transition lines

In order to divide the phase space into the different stability regions we had to analyze the transition lines that separate the following regions of dynamic behavior (Fig. S2):

1. The region of Turing instability occurs above the blue and green lines. In this region there are non-oscillatory unstable modes where: $\mathrm{Im}\{\omega_i\} = 0$ and $\mathrm{Re}\{\omega_i\} > 0$. The amplitude of these modes grow exponentially from small initial perturbations, but do not oscillate or propagate on the membrane surface.
2. The region of wave instability is bounded by the red and brown lines. In this region there are oscillatory unstable modes where: $\mathrm{Im}\{\omega_i\} \neq 0$ and $\mathrm{Re}\{\omega_i\} > 0$. The amplitude of these modes grow exponentially from small initial perturbations, and oscillate or propagate on the membrane surface.
3. The system is stable below the blue and red lines, such that initial perturbations decay exponentially; $\mathrm{Re}\{\omega_i\} < 0$. In this region there are damped waves to the right of the dashed green line, such that there are damped oscillatory modes with: $\mathrm{Im}\{\omega_i\} \neq 0$.

3.1 Turing instability transition

We are trying to find when there is a real eigenvalue with a positive sign. A Turing unstable region can be created in two ways:

3.1.1 Turing instability from a stable mode

In this case the real part of a stable mode crosses the x–axis. The transition lines is when $\mathrm{Re}\{\omega_i\} = 0$ for ω_i which is real ($\mathrm{Im}\{\omega_i\} = 0$). This transition line can be derived by finding the roots of $Det(L)$.

$$Det(L) = \begin{cases} \omega_1\omega_2\omega_3 & \omega_1, \omega_2, \omega_3 \in \mathbb{R} \\ \omega_1(x^2 + y^2) & \omega_1 \in \mathbb{R}, \quad \omega_2, \omega_3 \in \mathbb{C} \text{ and } \omega_2 = \omega_3^* \equiv x + iy \end{cases} \tag{S19}$$

The roots will determine when ω_1 is zero, without loss of generality. The zeros of the determinant mean that a real eigenvalue is zero. We want to find the first occurrence of such a crossing. Hence we will demand the crossing point to be a maximum, by looking at the derivative: $\partial \mathrm{Det}(L)/\partial q = 0$.

3.1.2 Turing instability from a unstable wave mode

Another way to transition to a state which has a real eigenvalue which is positive is if an unstable complex eigenvalue becomes real. For this case we looked at the following equations

$$\Delta(L) = 0 \text{ and } \partial\Delta(L)/\partial q = 0 \tag{S20}$$

where Δ is the discriminant of the characteristic polynomial of L. If the discriminant is positive a cubic equation will have all real roots and if the discriminant is negative there will be one real root and two imaginary ones. In this case we will want to find crossing point which are maxima.

3.2 Wave transition

The existence of an imaginary part for the eigenvalues is known to be determined by the sign of the discriminant of the characteristic polynomial of L. Therefore, the transition occurs when $\Delta(L) = 0$ (Δ is the discriminant). Again, in order to find the first change of sign we will demand that the point be a minimum, by looking at the derivative: $\partial\Delta(L)/\partial q = 0$.

3.3 Wave instability transition

This transition can arise in two ways. This transition line corresponds to the change of sign of the real part of a complex eigenvalue. Assuming

$$\omega_1 \in \mathbb{R}, \quad \omega_2, \omega_3 \in \mathbb{C} \text{ and } \omega_2 = \omega_3^* \equiv x + iy \tag{S21}$$

we would like to know when $\text{Re}\{\omega_2\}$ changes sign. $\text{Re}\{\omega_2\}$ is zero when the following polynomial

$$P \equiv Det(L) - Tr(L)\sum_n M_{nn} = -2x\left(x^2 + y^2 + 2x\omega_1 + \omega_1^2\right) = 0 \tag{S22}$$

where M_{nn} is the n-th principal minor. Over real space this polynomial is zero only when $\text{Re}\{\omega_2\} \equiv x = 0$. This assumes that the problem has complex eigenvalues hence the discriminant also needs to be negative ($\Delta(L) < 0$).

3.3.1 Far away from a Turing instability region

In this case we would like to find the first occurrence of such a transition (the Turing unstable region does not interfere), so we will find extremums by demanding that the derivative be zero: $\partial P/\partial q = 0$.

3.3.2 Adjacent to a Turing instability region

In this case the wave instability transition does not occur at an extremum of P but where two real eigenvalues are zero. In this case we looked into the set of equations $\text{Det}(L) = 0$ and $P = 0$ which can be simplified to $\text{Det}(L) = 0$ and $M_{nn} = 0$

4 Explanation of the phase diagrams in Fig. S2

The relation between A_- and A_+ along the Turing instability transition is linear in all the cases, with the slope proportional to: $\bar{\phi}_+/\alpha\bar{\phi}_-$, where $\alpha = |H_-|/H_+$. This shows that for Turing instability to occur the relative force due to the convex activators ($\propto |H_-|A_-\bar{\phi}_-$) needs to be larger than the relative force due to the concave activators ($\propto H_+A_+\bar{\phi}_+$).

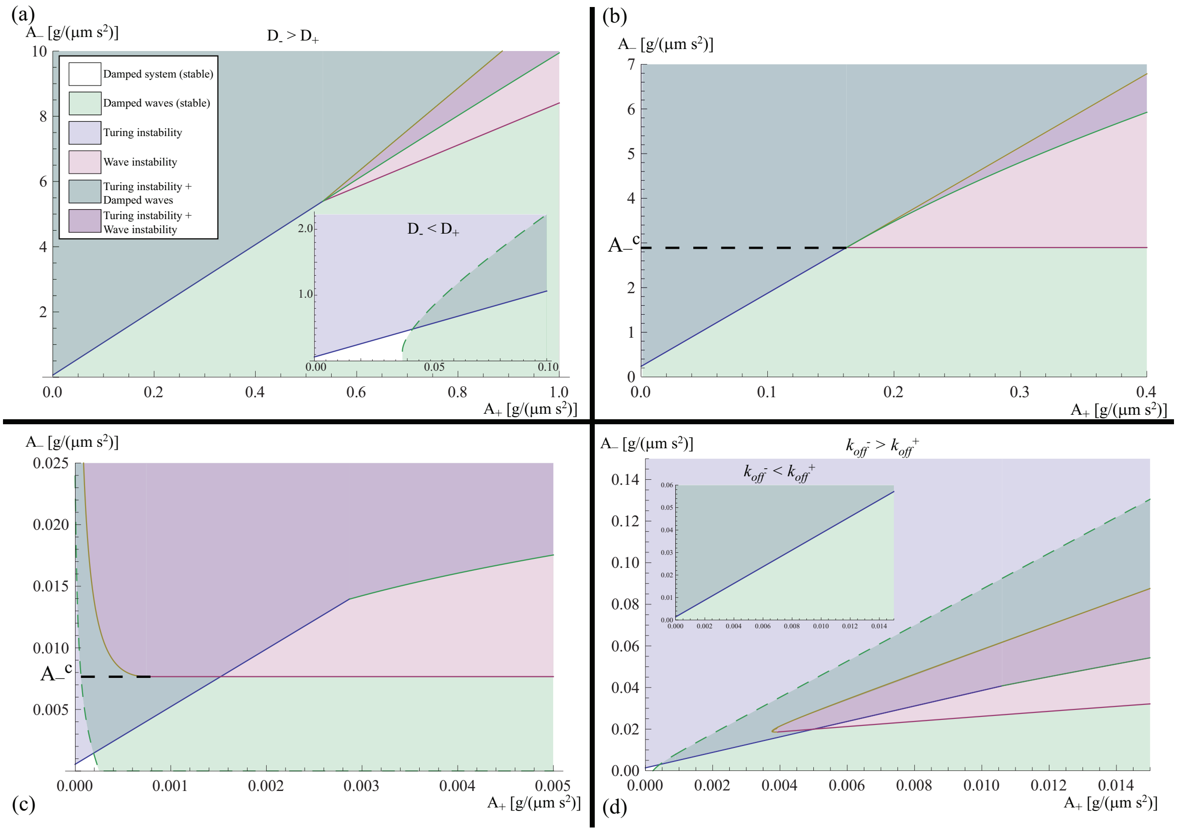

Figure S2: Stability phase diagram in the A_{-}–A_{+} plane. (a) the diffusion(-)–diffusion(+) model. The main figure depicts the phase diagram when $D_{-} > D_{+}$ while the inset depicts $D_{-} < D_{+}$. (b) the adsorption(-)–diffusion(+) model. (c) the diffusion(-)–adsorption(+) model. (d) the adsorption(-)–adsorption(+) model. The main figure depicts the phase diagram when $k_{\text{off}}^{-} > k_{\text{off}}^{+}$ while the inset depicts $k_{\text{off}}^{-} < k_{\text{off}}^{+}$. The legend correspond to all four subfigures. In subfigures (b) and (c) the threshold value of A_{-} is denoted by A_{-}^{c}.

4.1 Diffusion(-) – diffusion(+) model

The Turing instability transition line is given by

$$A_- = A_+ \frac{n_-^s \bar{\phi}_+}{\alpha n_+^s \bar{\phi}_-} + \sigma_{\text{eff}} \left(|H_-| + \frac{n_-^s T}{|H_-| \kappa \bar{\phi}_-} + \frac{H_+ n_-^s \bar{\phi}_+}{\alpha n_+^s \bar{\phi}_-} \right), \tag{S23}$$

where $\alpha = |H_-|/H_+$. The slope of the wave instability transition line is

$$\frac{1}{\gamma} \left(\frac{n_-^s \bar{\phi}_+}{\alpha n_+^s \bar{\phi}_-} \right) \left(\frac{n_-^s n_+^s \mathcal{O} T \sigma_{\text{eff}} + D_+ \left(n_-^s n_+^s T + H_-^2 n_+^s \gamma \kappa \bar{\phi}_- + H_+^2 n_-^s \kappa \bar{\phi}_+ \right)}{n_-^s n_+^s \mathcal{O} T \sigma_{\text{eff}} + D_+ \left(n_-^s n_+^s T \gamma + H_-^2 n_+^s \gamma \kappa \bar{\phi}_- + H_+^2 n_-^s \kappa \bar{\phi}_+ \right)} \right) \tag{S24}$$

where $\gamma = D_-/D_+$. We notice that the lines intersect in the first quadrant only when $\gamma > 1$ because the slope of the red line is smaller than the slope of the blue line. The intersection point is

$$A_- = \frac{D_- \left(n_-^s n_+^s T + H_-^2 n_+^s \kappa \bar{\phi}_- + H_+^2 n_-^s \kappa \bar{\phi}_+ \right) + n_+^s \mathcal{O} \sigma_{\text{eff}} \left(n_-^s T \gamma + H_-^2 (\gamma - 1) \kappa \bar{\phi}_- \right)}{|H_-| n_+^s \mathcal{O} (\gamma - 1) \kappa \bar{\phi}_-} \tag{S25}$$

$$A_+ = \frac{D_- \left(n_-^s n_+^s T + H_-^2 n_+^s \kappa \bar{\phi}_- + H_+^2 n_-^s \kappa \bar{\phi}_+ \right) + n_-^s \mathcal{O} \sigma_{\text{eff}} \left(n_+^s T - H_+^2 (\gamma - 1) \kappa \bar{\phi}_+ \right)}{H_+ n_-^s \mathcal{O} (\gamma - 1) \kappa \bar{\phi}_+} \tag{S26}$$

From this we understand that the stability behavior depends greatly on D_-/D_+. This can be seen in Fig. S2a. If $D_- < D_+$ then the model gives rise only to two types of behavior depending on the values of the parameters A_- and A_+: (i) Turing instability above the blue line and/or, (ii) damped waves, below the green line (Inset of Fig. S2a). The system does not exhibit a behavior of unstable waves. The system is stable below the blue line. This transition line is linear in A_+ and σ_{eff}, and given in Eq. S23.

In the region of the phase diagram which displays both Turing instability and damped waves (dark green region), these two behaviors appear for the same wavevector but in two different eigenmodes of the system (see SI section 6). This emphasizes that in this system the oscillatory and unstable behaviors are completely separated. Note that the slopes of the Turing and damped waves transition lines become parallel for large A_- and A_+ when the diffusion coefficients of the two activators are equal. Only if $D_- < D_+$ the model also exhibits an unstable wave behavior in a wedge region (colored pink and purple in Fig. S2a).

4.2 Adsorption(-) – adsorption(+) model

The phase diagram is shown in Fig. S2d. The Turing instability transition line is given by:

$$A_- = A_+ \frac{n_-^s \bar{\phi}_+}{\alpha n_+^s \bar{\phi}_-} + \sigma_{\text{eff}} \frac{n_-^s T}{2|H_-| \kappa \bar{\phi}_-} \tag{S27}$$

and has the same slope as in the diffusion(-)–diffusion(+) model. Waves only occur for $\eta \equiv k_{\text{off}}^-/k_{\text{off}}^+ > 1$ and

$$A_+ > \frac{n_+^s T \sigma}{2H_+ (\eta - 1) \kappa \bar{\phi}_+} + \sqrt{\frac{k_{\text{off}}^- n_+^s T \left(n_+^s T + 2H_+^2 (\eta - 1) \kappa \bar{\phi}_+ \right)}{H_+^2 \mathcal{O} (\eta - 1)^2 \kappa \bar{\phi}_+^2}} \tag{S28}$$

The stability behavior depends significantly on $k_{\text{off}}^-/k_{\text{off}}^+$. This can be seen in Fig. S2d. If $k_{\text{off}}^- < k_{\text{off}}^+$ the model gives rise to two types of behaviors: Turing instability and damped waves (Inset of Fig. S2d). The system is stable below the Turing instability transition line and the transition is defined by Eq. S27.

In the region of the phase diagram which displays both Turing instability and damped waves (dark green region), these two behaviors appear for the same wavevector but in two different eigenmodes of the system (see section 6). This emphasizes that in this system the oscillatory and unstable behaviors are completely separated. If $k_{\text{off}}^{-} > k_{\text{off}}^{+}$ the model also has a wave instability region in the phase diagram (colored pink and purple). This region is bounded by both the red line and brown line. The Turing instability transition occurs either by the changing of sign of the determinant or by the splitting of the unstable wave region into two creating a Turing instability region between the two (see SI section 6).

4.3 Diffusion(-) – adsorption(+) model

In this model we found a much more diverse set of behaviors as is depicted in Fig. S2c. We found a region in parameter space where the system has a wave instability (above the red line in Fig. S2c). The Turing instability transition line has two regimes (see section 8 for further details). The physically relevant condition is marked in blue in Fig. S2c and is linear in A_+ and σ_{eff}

$$A_- = \left(A_+ \frac{2\bar{\phi}_+}{\alpha\bar{\phi}_-} + \sigma_{\text{eff}} \frac{n_+^s T}{|H_-|\kappa\bar{\phi}_-} \right) \frac{n_-^s T + H_-^2 \kappa\bar{\phi}_-}{n_+^s T - 2H_+^2 \kappa\bar{\phi}_+}, \tag{S29}$$

The parameters that control the adsorption kinetics of the activator, k_{off}, μ, affect the phase diagram. This can be seen for example in the expression for the Turing instability transition (Eq. S29) in the parameter $\bar{\phi}_+ = \exp\left[\mu - \kappa H_+^2 / n_+^s T\right]$.

In this case, the region of the phase diagram which displays both a Turing instability and damped waves behavior exhibits these two behaviors for the same eigenmode but at different wavevectors (see section 6). The system is stable in the region of the phase diagram below both the blue and the red lines. The waves and wave instability transition lines are calculated numerically. Changing the parameters to the two extremes of mobility $D_-/\sqrt{\mathcal{O}\kappa k_{\text{off}}} \ll 1$ or $D_-/\sqrt{\mathcal{O}\kappa k_{\text{off}}} \gg 1$ does not change the phase diagram qualitatively as in the diffusion(-)–diffusion(+) and adsorption(-)–adsorption(+) models. It does not eliminate the wave instability region.

The wave-instability transition line is only weakly dependent on A_-, for small A_+. This model exhibits a threshold like value for A_- above which unstable waves occur for most values of A_+ . This threshold is given by:

$$\begin{aligned} A_-^c \quad \simeq \quad & \frac{1}{|H_-|\bar{\phi}_-\left(n_-^s T + H_-^2 \kappa\bar{\phi}_-\right)^2} \left(\frac{\sigma_{\text{eff}}\left(n_-^s T + H_-^2 \kappa\bar{\phi}_-\right)^3}{\kappa} + \frac{k_{\text{off}} n_-^s \left(n_-^s T + H_-^2 \kappa\bar{\phi}_-\right)\left(2n_-^s T + H_-^2 \kappa\bar{\phi}_-\right)\left(n_+^s T - 2H_+^2 \kappa\bar{\phi}_+\right)}{D_- n_+^s} \right) \\ + \quad & \frac{2}{|H_-|\bar{\phi}_- D_- n_+^s} \\ \times \quad & \sqrt{\frac{k_{\text{off}}}{\mathcal{O}\kappa\left(n_-^s T + H_-^2 \kappa\bar{\phi}_-\right)} n_-^s T \left(D_-^2 {n_+^s}^2 \left(n_-^s T + H_-^2 \kappa\bar{\phi}_-\right)^2 + D_- n_-^s n_+^s \mathcal{O}\sigma_{\text{eff}} \left(n_-^s T + H_-^2 \kappa\bar{\phi}_-\right)\left(n_+^s T - 2H_+^2 \kappa\bar{\phi}_+\right) + k_{\text{off}} {n_-^s}^2 \mathcal{O}\kappa \left(n_+^s T - 2H_+^2 \kappa\bar{\phi}_+\right)^2 \right)} \end{aligned} \tag{S30}$$

4.4 Adsorption(-)–diffusion(+) model

The Turing instability transition line is given by:

$$A_- = A_+ \frac{\left(H_+ n_-^s T \kappa\bar{\phi}_+ - 2H_-^2 H_+ \kappa^2 \bar{\phi}_- \bar{\phi}_+\right)}{2|H_-| n_+^s T \kappa\bar{\phi}_- + 2|H_-| H_+^2 \kappa^2 \bar{\phi}_- \bar{\phi}_+} + \sigma_{\text{eff}} \frac{\left(n_-^s n_+^s T^2 + H_+^2 n_-^s T \kappa\bar{\phi}_+\right)}{2|H_-| n_+^s T \kappa\bar{\phi}_- + 2|H_-| H_+^2 \kappa^2 \bar{\phi}_- \bar{\phi}_+} \tag{S31}$$

Unstable waves occur above a threshold value of A_- given by

$$A_-^c = \frac{n_-^s \left(n_+^s T (D_+ + \mathcal{O}\sigma_{\text{eff}}) + D_+ H_+^2 \kappa\bar{\phi}_+\right)}{2|H_-| n_+^s \mathcal{O}\kappa\bar{\phi}_-} \tag{S32}$$

which is independent of A_+. As in the diffusion(-)–adsorption(+) model, changing the parameters to the two extremes of mobility $D_+/\sqrt{\mathcal{O}\kappa k_{\text{off}}} \ll 1$ or $D_+/\sqrt{\mathcal{O}\kappa k_{\text{off}}} \gg 1$ does not change the phase diagram qualitatively as in the diffusion(-)–diffusion(+) and adsorption(-)–adsorption(+) models. It does not eliminate the wave instability region.

5 Simplified diffusive(-)–adsorptive(+) model

We can simplify the model using the following assumptions:

1. The active force of actin polymerization is larger than the passive force with which each activator bends the membrane.

2. There is no direct interaction between the two activators.

Furthermore, in order to get an approximation for the the most unstable wavenumber, we took the highest orders of q of the polynomial $P = Det(L) - Tr(L)\sum_n M_{nn}$ where M_{nn} is the n-th principal minor. The zeros of this polynomial can be solved analytically and we receive the following approximation for the most unstable wavenumber:

$$q_{\text{approx}} \approx \sqrt{\left(\frac{-D_-^2 n_-^s n_+^s \mathcal{O}T - k_{\text{off}}^+ n_-^s n_+^s \mathcal{O}^2 T\kappa + A_- D_- |H_-| n_+^s \mathcal{O}^2 \kappa \bar{\phi}_-}{2D_- \left(k_{\text{off}}^+ \kappa\right)^{1/2} n_-^s n_+^s T\mathcal{O}^{3/2}} + \frac{\sqrt{n_+^s \mathcal{O}^2 \left(n_+^s \left(n_-^s T\left(D_-^2 + k_{\text{off}}^+ \mathcal{O}\kappa\right) - A_- D_- |H_-| \mathcal{O}\kappa \bar{\phi}_-\right)^2 + 4D_- n_-^s \mathcal{O}T\kappa (D_- n_+^s (-2k_{\text{off}}^+ n_-^s T + A_- D_- |H_-| \bar{\phi}_-) - 2A_+ H + k_{\text{off}}^+ n_-^s \mathcal{O}\kappa \bar{\phi}_+)\right)}}{2D_- \left(k_{\text{off}}^+ \kappa\right)^{1/2} n_-^s n_+^s T\mathcal{O}^{3/2}} \right)} \tag{S33}$$

6 Examples of dispersion relations

We give in Fig. S3 several typical dispersion relations that arise in our model, to illustrate the different behaviors that appear in the regions with the corresponding color in the phase diagrams (Fig. S2); (a) represents purely real damped modes (white), (b) represents damped wave modes (light green), (c) represents pure Turing instability (blue), (d) case where there are Turing instability and damped waves but in different eigenmodes (dark green), (e) as in (d) but for the same eigenmode (dark green), (f) pure wave instability (pink), (g) wave instability at the edge of the Turing instability region (purple) and (h) Turing instability bounded by regions of wave instability (purple).

7 Method of holding the membrane

In the derivation presented in the article, we moved to the moving frame of the membrane in order to keep the membrane displacement from growing all the time. It is also possible to add an osmotic pressure term to the free energy of the form:

$$\mathcal{F}_p = \int_S \frac{P}{\tilde{l}} h^2 \sqrt{1 + (\nabla h)^2} d^2 r \tag{S34}$$

where P is the osmotic pressure and $\tilde{l}$ is the unit length of the symmetric coordinate. Again, keeping terms only quadratic in h, we arrive at

$$\mathcal{F}_p = \int_S \frac{P}{\tilde{l}} h^2 d^2 r \tag{S35}$$

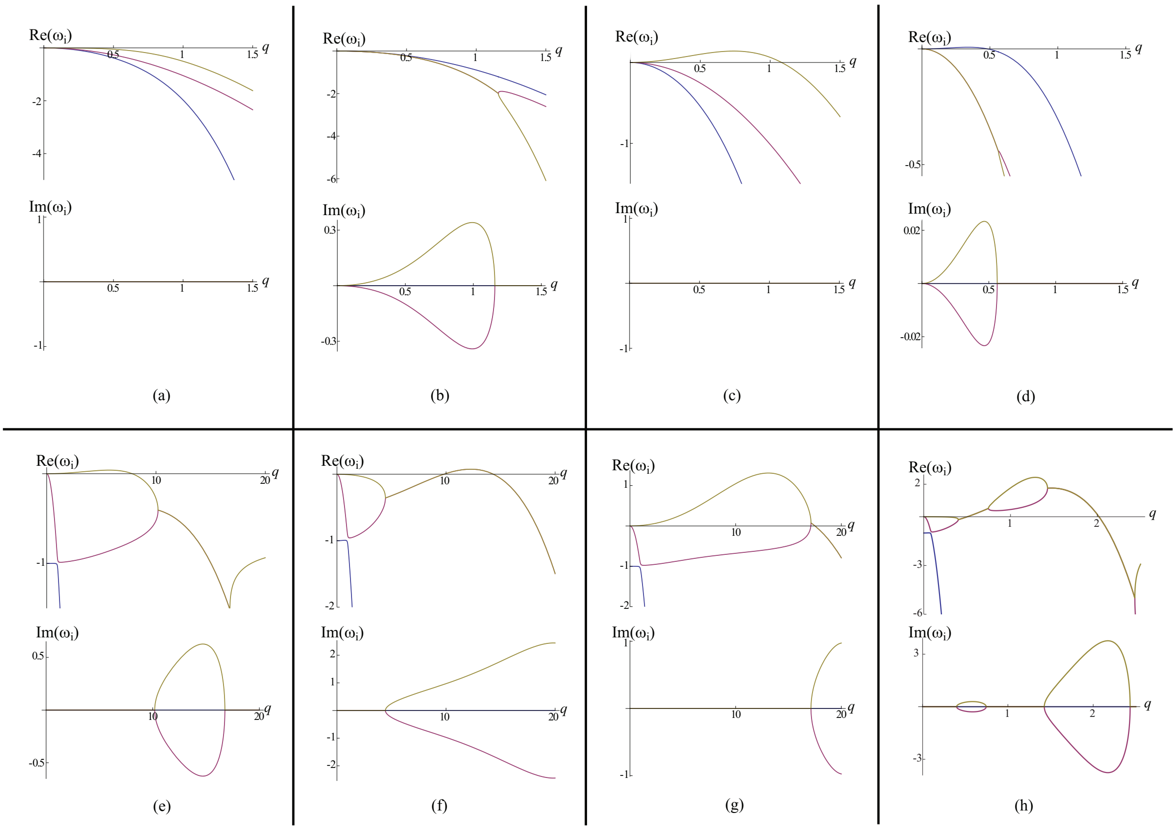

Figure S3: Dispersion relations: (a) Damped system (stable). (b) Damped waves (stable). (c) Turing instability (d-e) Turing instability and damped waves. (d) Turing instability and damped waves in two different modes of the system. (e) Turing instability and damped waves in the same mode of the system. (f) Wave instability away from a Turing instability area. (g) Wave instability adjacent to a Turing instability region. (h) Turing instability created from a wave instability region.

This adds a term of the form $-\mathcal{O}Ph$ to the membrane's displacement equation of motion which is a restoring force. Linear stability analysis and nonlinear simulations done with this added term result in qualitatively identical behaviors.

8 Turing instability transition line in the diffusion(-)–adsorption(+) model

Studying the condition where $Det(L) = 0$ for real wavevectors results in the following conditions:

$$\begin{cases} A_- \geq 0 & \text{for } \kappa > \frac{n_+^s T}{2H_+^2 \bar{\phi_+}}, \\ A_- > \left(A_+ \frac{2\bar{\phi_+}}{\alpha \bar{\phi_-}} + \sigma_{\text{eff}} \frac{n_+^s T}{|H_-| \kappa \bar{\phi_-}}\right) \frac{n_-^s T + H_-^2 \kappa \bar{\phi_-}}{n_+^s T - 2H_+^2 \kappa \bar{\phi_+}} & \text{for } 0 < \kappa < \frac{n_+^s T}{2H^2 \bar{\phi_+}} \end{cases} \tag{S36}$$

The first condition occurs in a regime where the adsorption of the second protein is very large. In this case the system is most unstable at the shortest wavelength possible (There is some cutoff which depends on the thickness of the membrane and the size of the protein). This regime's condition, once $\bar{\phi_+}$ is substituted, becomes:

$$\frac{\kappa H_+^2}{n_+^s T} > \frac{1}{2} \exp\left(\frac{\kappa H_+^2}{n_+^s T} - \mu\right) \tag{S37}$$

This condition can be satisfied only for large positive values of μ. The minimal value of μ is given when $\kappa H_+^2/(n_+^s T) = 0$, i.e. $\mu > 1 - \ln(2) \approx 0.307$. In the biologically relevant system we expect μ to be negative because the activators have an affinity to the membrane, making this regime irrelevant. Furthermore, we expect that realistically the concentration of $\bar{\phi_+}$ is small, hence making the regime where this condition applies occur only for values of κ which are not biologically relevant. In the main text we refer only to the second regime.

9 Non-linear membrane tension

We add a non-linear tension term because of the finiteness of the membrane size, which limits the amplitude of the membrane height deformation in the CDR. This amplitude can be estimated to be of order 1μm from confocal images of CDRs, as shown in Fig. S4.

As can be seen in Fig. S5 the non-linear tension does not affect strongly the wave velocity in our calculations, and only limits the membrane height displacement as is seen in the mean square amplitude of the membrane.

When β is small, the amplitude of the membrane shape is such that the linear Monge description of the curvature is inaccurate. Note that by limiting the growth of the membrane fluctuations, we prevent strong depletions of the concentration distributions of the activators, which would result in a significant slowing down of our simulations.

When the parameters correspond to a region that has both wave and Turing instabilities, a larger value of the non-linear tension prevents the Turing steady-state from dominating.

10 Turing instability

In Fig. S6 we show the calculation of the system corresponding to a point in Fig. S2a (diffusion(-)–diffusion(+) system) given by the values: $A_- = 0.5$ g/(μm s^2), $A_+ = 0$. We find that indeed the most unstable mode grows the fastest, but eventually the individual membrane protrusions coalesce to form a single protrusion (a similar process was shown in [2]). This simulation is shown in Supporting Movie

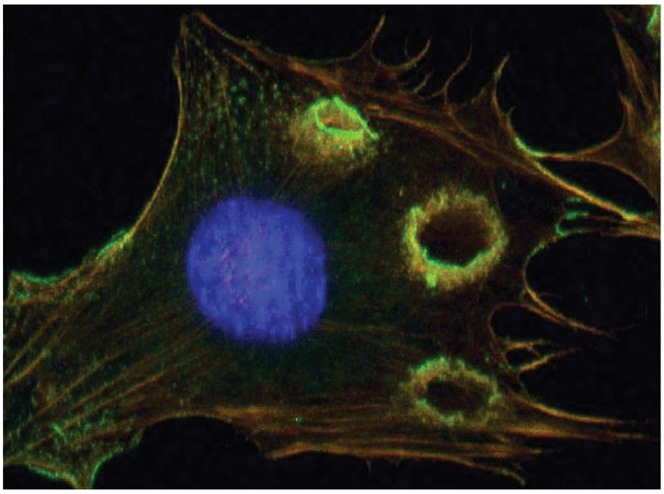

Figure S4: Experiment done in MEF cells which are stimulated by PDGF. A three dimensional reconstruction of 3 ruffles. Eps8 is marked in green and actin is marked in red.

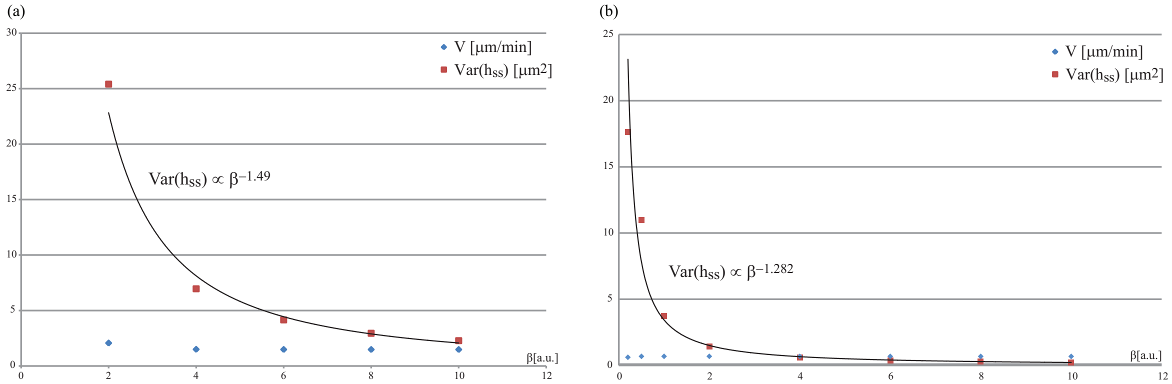

Figure S5: Mean square steady state membrane amplitude power law dependence and group velocity independence of the non-linear tension coefficient β. (a) Diffusion(-)–diffusion(+) model. (b) Diffusion(-)–adsorption(+) model.

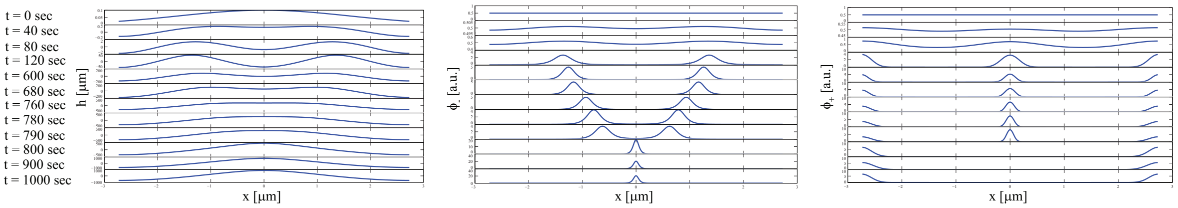

Figure S6: Time dependence of the membrane height displacement and activator concentrations. In the early times, the most unstable wavelength appears while the steady state is a single protrusion.

4. Note that the amplitude of the membrane protrusions shown in Fig. S6 is so large, that the Monge representation used here and the linear equations for the membrane shape, are not accurate anymore. The calculation is therefore an approximation of the real dynamics and illustrates the qualitative nature of the evolution of the protrusions.

References

[1] Gov N, Zilman A, Safran S (2004) Hydrodynamics of confined membranes. Physical Review E 70: 011104.

[2] Shlomovitz R, Gov NS (2009) Membrane-mediated interactions drive the condensation and coalescence of ftsz rings. Physical biology 6: 046017.
